# Can serum procalcitonin measurement help monitor the treatment of acute bacterial meningitis? A prospective study

**Published:** 2012

**Authors:** Seyed Mohammad Alavi, Shahram Shokri

**Affiliations:** 1 Joundishapour Infectious and Tropical Diseases Research Center, Joundishapour University of Medical Sciences, Ahvaz, Iran.; 2 Department of Infectious Diseases, Razi Hospital, Joundishapour University of Medical Sciences, Ahvaz, Iran.

**Keywords:** Procalcitonin, Acute bacterial meningitis, Antibacterial treatment.

## Abstract

**Background:**

Previous studies have demonstrated the value of serum procalcitonin (PCT) as a marker of bacterial infection, as well as the rapid decrease in its concentration with appropriate antibiotic treatment. The aim of this study was to determine the variation in serum PCT levels over time during the treatment of acute bacterial meningitis (ABM) in adults.

**Methods:**

In this prospective analytical study, 36 acute meningitis patients (26 males and 10 females) with mean age of 38.4±20.1 years were studied. Among them, 17 patients with fever and neck stiffness and CSF profiles consistence, ABM was treated by appropriate antibiotic regimen. We measured PCT serum levels before and after 24 and 72 hours after initiation of treatment. Decrease in the body temperature and feeling of well being were the clinical criteria for treatment response. The data were collected and analyzed.

**Results:**

Serum level of PCT in the beginning of treatment, 24 and 72 hours after initiation of treatment was 2.58±4.62, 2.50±4.6 and 1.52±3.03 ng/ml, respectively. Difference in PCT initially and 24 h later was 0.044±0.08 (p=0.025) and after 72 h was 1.74±2.92 (p=0.013). The mean of PCT level was greater in patients not improving (5.34±4.42 vs. 2.08±3.46).

**Conclusion:**

Although decreasing PCT was low in 24 h after treatment, this decrement is more significant after 72 h. PCT serum level may be used as a follow up of treatment response in ABM.

Acute bacterial meningitis (ABM) is a medical emergency and remains a serious disease with mortality rates of 10–25% (1). Prompt antibiotic treatment improves the outcome of ABM. For patients with suspected meningitis, immediate lumbar puncture (LP) before antibiotics is recommended to maximize the chance of a positive cerebrospinal fluid (CSF) culture (2). Early differentiation between ABM and aseptic meningitis (ASM) is of central importance for antibacterial therapeutic decision-making. Although the patients with ASM may not require antibiotic therapy, immediate administration of antibiotics is essential to improve survival in patients with ABM (3). Appropriate antibacterial treatment should be started rapidly when the diagnosis of ABM has been established, particularly when the risk factors are present (4, 5). 

The choice of antibiotic treatment is based on age, risk factors, and the results of CSF examination and knowledge of bacterial ecology (3). As a routine, the efficacy of this initial antibiotic therapy is assessed by clinical improvement and analysis of cerebrospinal fluid (CSF) samples obtained 48–72 hours after the start of treatment when available, although cytochemical CSF parameters appear to be little modified by appropriate antibiotic treatment (1). Due to the patient’s avoidance against repeated lumbar puncture (LP), follow up CSF analysis encountered some difficulties. Thus, monitoring of ABM treatment is a challenging subject for physicians who are involved in ABM management.

To find a way for evaluating the ABM treatment without the need of repeating LP was a problem engaging in our minds for many years. A marker that could demonstrate efficacy at an earlier stage would be extremely useful. Until recently, no laboratory marker has been available to be a substitute for repeated CSF examination to monitor ABM treatment. Previous studies have demonstrated the value of serum procalcitonin (PCT) as a marker of infectious states of bacterial origin in children, as well as the rapid decrease in its concentration with appropriate antibiotic treatment (3, 6). Ray et al. reported that PCT is a useful tool for differentiating ABM from ASM (7). There is limited data about the value of PCT in Iran. The aim of the present study was to determine the variation in serum PCT levels over time during the treatment of ABM.

## Methods

All the patients admitted to the adult section at the Department of Infectious Diseases with acute meningitis from January 2008 to October 2009 were studied. The demographic and clinical characteristics of the patients were recorded on admission. Most of our patients after having received antibiotics for at least 2 days before hospitalization, ABM was diagnosed as probable when polymorphonuclear leukocyte count in the CSF exceeded 100/mm^3 ^and the CSF/serum glucose ratio was below 0.4, with a compatible clinical state such as fever and neck stiffness (3). ABM was confirmed if CSF examination for bacterial evidence (smear or culture) was positive. PCT was positive if a serum PCT level was 0.5 ng/ml or more. The patients with clinical criteria and CSF pleocytosis but lacking ABM criteria were considered as ASM. The patients presented with a further site of infection in addition to meningitis on admission, having a minimum age of 14 or less, and severe underlying diseases were excluded from the study.

**Laboratory Examinations: **Blood samples for PCT, blood sugar and complete blood count were taken on admission. Lumbar puncture (for total and polymorphonuclear leukocyte count and assay of proteins and glucose) and bacteriological sampling (blood cultures) were performed before starting the initial antibiotic treatment. These tests were repeated 24 and 72 hours after initiation of treatment. The interval between admission and administration of the first dose of antibiotic was recorded. Serum PCT levels were determined using a quantitative Elecsis method with commercial kit (Brahms, Germany) with a cut off detection of 0.5 ng/ml. 

**Treatment and Outcome: **As a routine, our first choice empirical antibacterial regimen for ABM was intravenous Ceftriaxon plus Vancomycin based on patient’s body weight. In cases with age of more than 65 years, Ampicilin was added (3). CSF was assessed in all patients in 24 and 72 hours after initiation of treatment.

The efficacy of administered antibiotic was assessed on the basis of cytochemical analysis of CSF samples (PMN percent decrement and CSF/serum glucose ratio rising) in 24 and 72 hours after treatment initiation, and clinical course. Decrease in body temperature and feeling of well being were the clinical criteria for treatment response.

**Statistical analysis: **Results are expressed as mean±standard deviation. The data were analyzed in SPSS, 16 software for windows using t-test for quantitative parameters (mean PCT serum level) and the chi-square test for qualitative parameters (the percentage of patients having PCT positive tests in studied patients), with the threshold of significance set at p<0.05. We also used independent sample t-test to compare the serum level of PCT with clinical improvement.

## Results

During the study period (12 months), 48 patients presented with acute meningitis were admitted to the Infectious Disease Ward. Twelve patients were excluded for the following reasons: age below 14 years (n=4), presence of another site of infection (urosepsis, n=2; pneumonia, n=1), and underlying diseases (diabetes mellitus, n=3; HIV infection, n=2). From the total 36 cases with acute meningitis, 17 were ABM and 19 were ASM. Among the 17 CSF samples, 12 samples were positive for bacterial evidence (blood culture, CSF gram stain & cultures). Out of the 36 patients, 26 (72.2%) were males and 10 (27.8%) were females with mean age of 38.4±20.1 years.

**Figure 1:**
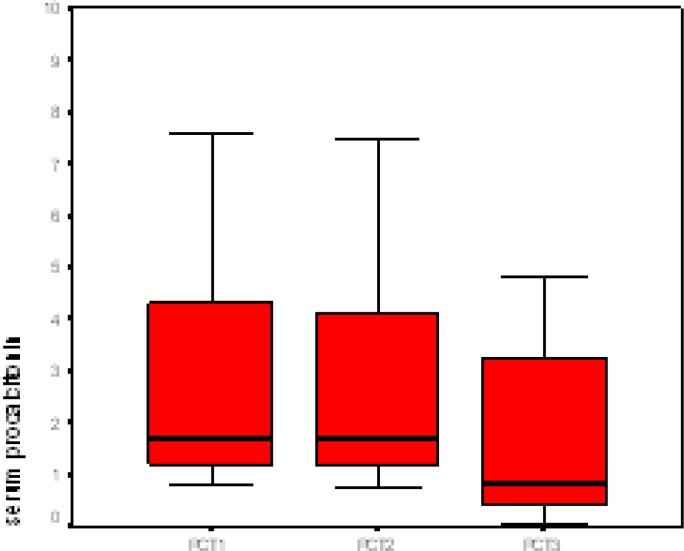
Distribution of serum procalcitonin among patients with bacterial meningitis before and after empirical antibiotic treatment

In the 36 patients who underwent a repeat lumbar puncture, the duration of antibiotic treatment (in 17 ABM patients) was 14–21 days, resulting in cure in 15 (88.2%) based on clinical improvement and CSF cytochemical changes. Cure rate in ASM (without antibiotic treatment) was 94.7% (18, 19). The duration of hospital stay for ABM cases was between 14 and 28 days and for ASM was 7-10 days. The decrease in PCT level and PMN percent was the only significant difference observed between time of admission and 72 hours after treatment (among ABM patients) in serum parameters. There was no significant correlation (p=0.19) between CSF pattern and PCT serum level. From the 36 patients, 29 (80.6%) have received antibiotics before hospitalization (table 1). Serum level of PCT in the beginning of treatment, 24 and 72 hours after initiation of treatment was 2.58±4.62, 2.50±4.6 and 1.52±3.03 ng/ml, respectively (p=0.013, p=0.025), whereas, CSF cytochemical parameters showed no considerable changes, 24 and 72 hours after treatment. There was significant decrease in serum PCT after empirically treatment of ABM. Distribution of serum PCT in patients with ABM before and after empirical antibiotic treatment is shown in figure 1.

**Table 1:** Comparison of positive and negative results of serum procalcitonin among patients with acute meningitis based on CSF cytochemical analysis and previous received antibiotic

**pvalue**	**Serum PCT in admission**		
	**Negative**	**Positive**	
0.192	5 10	12 9	CSF pattern PLG MNG
0.674	13 2	16 5	Previous antibiotic Received Not received

CSF: cerebrospinal fluid, PLG: polymorphonuclear predominance and low CSF: serum glucose ratio, MNG: mononuclear predominace and normal CSF glucose level. PCT: procalcitonin

PCT1: PCT before treatment, PCT2: PCT 24 h after treatment, PCT3: 72 h after treatment

## Discussion

This study showed that early decrease in serum level of PCT was significantly associated with cure of ABM. In contrast, analysis of CSF showed a non significant decrease in CSF-cytochemical parameters between 48 and 72 hours after antibacterial treatment. Similar findings were reported by earlier investigators concerning the changes in serum PCT concentration after 24–72 hours of treatment for ABM (1, 8). Viallon et al. reported a significant relation between PCT serum level and clinical course of ABM (1).

Although previous studies have explained that PCT serum level decrease following antibacterial treatment, few data are available concerning the change in serum PCT during treatment for ABM (6, 9-11). Schwartz et al. reported a reduction in serum PCT concentration after 48 hours of treatment in patients with ABM (10). 

Gendrel et al. reported that serum PCT concentration diminished within 24 hours of treatment in 81.8% children receiving treatment for ABM (11). In the present study, there was no significant decrease in the leukocyte count or protein concentration in the CSF after 24–72 hours of appropriate antibiotic treatment. The glucose concentration measured in the CSF remained stable. The earlier studies and literature describe that the CSF cytochemical parameters traditionally during ABM are little modified by appropriate antibiotic therapy within 48 hours (6, 12, 13). The present study revealed a rapid decrease in PCT concentration within 24-72 hours of treatment, which was accompanied by clinical improvement in a majority of our patients after 2–3 days. We did not have a control group not receiving appropriate treatment (based on medical ethics). How do we support the relationship between decrease in PCT levels and appropriate antibiotic treatment? We are unable to answer this question but in a study, an author has investigated the value of PCT serum level in relation with the initial antibiotic therapy in 43 patients presenting with melioidiosis of various grades of severity (14). 

There are some limitations in this study that should be mentioned. This was a descriptive study of the changes in serum PCT concentrations before and after treatment in patients with ABM who had received appropriate antibiotic treatment when they were admitted to the hospital. We currently have no data on changes in serum PCT levels occurring in patients who did not receive appropriate treatment. The ABM patients with a serum PCT below 0.5 ng /ml on admission were not included in our analysis. At present, there is no clear explanation for this finding. Although decreasing PCT was low in 24 h after treatment, this decrement is more significant after 72 h. PCT serum level may be used as a follow up treatment response in acute bacterial meningitis.
